# Multilateral Use of Dandelion in Folk Medicine of Central-Eastern Europe

**DOI:** 10.3390/plants14010084

**Published:** 2024-12-30

**Authors:** Robert Gruszecki, Magdalena Walasek-Janusz, Gianluca Caruso, Robert Pokluda, Alessio Vincenzo Tallarita, Nadezhda Golubkina, Agnieszka Sękara

**Affiliations:** 1 Department of Vegetable and Herb Crops, Faculty of Horticulture and Landscape Architecture, University of Life Sciences in Lublin, 20-280 Lublin, Poland; robert.gruszecki@up.lublin.pl; 2 Department of Agricultural Sciences, University of Naples Federico II, Portici, 80055 Naples, Italy; gcaruso@unina.it (G.C.); lexvincentall@gmail.com (A.V.T.); 3 Department of Vegetable Sciences and Floriculture, Faculty of Horticulture, Mendel University in Brno, Valticka 337, 691 44 Lednice, Czech Republic; robert.pokluda@mendelu.cz; 4 Federal Scientific Center of Vegetable Production, Selectsionnaya 14, VNIISSOK, Odintsovo District, 143072 Moscow, Russia; segolubkina45@gmail.com; 5 Department of Horticulture, Faculty of Biotechnology and Horticulture, University of Agriculture, 31-120 Kraków, Poland; agnieszka.sekara@urk.edu.pl

**Keywords:** *Taraxacum* sect. *Taraxacum*, pharmacological activity, traditional folk medicine, herbal medicine products

## Abstract

Background: Dandelion (*Taraxacum* sect. *Taraxacum*, also referred to as *Taraxacum officinale* F.H. Wiggers coll.), a collective species of perennial herbaceous plants of the Asteraceae family, is commonly considered weed; however, in the traditional societies of Central-Eastern Europe, it is a source of food and medicinal raw materials. The growing interest in the medicinal properties of herbal raw materials of dandelion encouraged us to focus on their use in the traditional folk medicine of Central-Eastern European communities. Aim: The hypothesis of the present study suggests that *Taraxacum* sect. *Taraxacum* (dandelion), which is widespread throughout Central-Eastern Europe and easily identifiable, has had notable applications in ethnopharmacology. The study aims to examine the medicinal properties of this species, focusing on its traditional uses in folk medicine across the region. The resulting data may serve as a valuable resource for contemporary pharmacognosy research. Methods: The analysis was based on publications dated from the end of the 18th century to the beginning of the 21st century, mainly from Poland. In addition, the study includes publications on contemporary Belarus, Ukraine, and European Russia. Results: The research showed that dandelion provided many medicinal raw materials, but the available literature did not mention the use of the herb’s root, a raw material popular in contemporary natural medicine. During the period analysed, an increase in the types of raw materials was observed, and the order in which they appeared in the sources was as follows: latex > root = leaf = inflorescence > herb > herb with flowers. Additionally, a review of the literature indicated that the number of conditions under which they were used increased during the investigation period. The variety of ways to prepare the raw material is noteworthy; fresh and dried raw materials were used to make extracts, tinctures, decoctions, infusions, wrap compresses, syrups, and even wine or coffee substitutes. The mentioned preparations were prepared individually for particular ailments. Conclusions: The vast experience of rural communities in Central-Eastern Europe concerning the medicinal applications of common dandelion has developed through centuries. The experience of rural communities may influence the direction of further phytochemical and pharmacological research.

## 1. Introduction

Common dandelion (*Taraxacum* sect. *Taraxacum*, also referred to as *Taraxacum officinale* F.H. Wiggers coll.) is a collective species of perennial herbaceous plants of the Asteraceae family living throughout mild climates of the northern hemisphere and is widely recognised as a weed. Eastern Slavs named this flower ‘the golden eye of God’ or ‘tiny sun’ due to easily recognisable yellow inflorescences and silver-tufted, wind-dispersed achenes. Yellow inflorescences in full blooming stage were a sign that man was a child of God and should be as good as the Sun. Single inflorescences at the end of the blooming period indicated that there were much fewer good people and their lives were often bitter, like the taste of the dandelion latex [1]. For the rural communities of Central-Eastern Europe, dandelion was not only a colourful prairie plant and weed of the farmlands but also a supplementary food during famine, an ingredient in salads, and the raw material for the preparation of syrup, coffee, beer, and wine [2,3,4,5,6,7,8,9,10,11,12,13,14]. The population of the mentioned region has never underestimated the medicinal properties of dandelion, and, moreover, folktales refer to the medicinal uses of dandelion, e.g., in the treatment of eye problems (blindness) with latex or of warts with ‘wart’ [6,15,16,17,18,19]. The Latin name, *Taraxacum officinale*, also indicates its healing properties, derived from the Greek ‘taraxis’ that means ‘inflammation’ and ‘akeomai’ meaning ‘healing’, while ‘*officinale*’ means ‘medicinal’ emphasising its therapeutic effects [20]. The curative effects of dandelion in folk tradition were included in collective elaborations covering these issues [6,18,21], although monographs of this species were not comprehensive, taking into account its widespread occurrence and use in folk medicine.

The increasing number of studies published in recent years revealed the healing properties of dandelion, including antiviral, e.g., against the SARS-CoV-2 virus [22,23,24,25]. The main hypothesis of the present study is that common dandelion, which is widespread in Central-Eastern Europe and easily identifiable due to its large yellow inflorescences, should be widely used in folk medicine. The aim of this study is to analyse the use of dandelion’s raw materials by the rural communities inhabiting Central-Eastern Europe in order to present their possible applications in contemporary medicine. The collection and analysis of information from Slavic-language literature on the use of dandelion’s raw material in the traditional medicine of the communities native to the mentioned regions is difficult for English-speaking scientists. However, it provides many novel links to the therapeutic applications of dandelion and indicates the direction of further research related to its use in modern medicine.

## 2. Materials and Methods

The analysis of the medicinal use of the common dandelion (*Taraxacum* sect. *Taraxacum*) in folk medicine of Central-Eastern Europe was carried out on the basis of printed studies including available elaborations since the 18th till 20th century, covering Central-Eastern Europe, with the particular emphasis on Poland, Belarus, European Russia, and Ukraine. The criticism method proposed by Topolski [26] was utilised in this study to analyse historical written sources and to evaluate the reliability of informants and information. External and internal source criticism was applied to achieve the proper interpretation of the source text and to verify the authenticity of the information provided by the authors based on the psychological and sociological background, as well as former social and physical environment [27]. The analysis was based on the database and card catalogue maintained by the ‘Pracownia Etnolingwistyczna’ at Maria Curie-Skłodowska University in Lublin. The results presented in this study are derived from 66 publications pertaining to the examined region, while 37 additional works from other global regions were included for comparative analysis. Only original and popular science publications were considered. In the process of source selection, folk names of *Taraxacum* (dandelion) were referenced according to the works of Annenkov [19] and Hyrcyna [18]. The following criteria were applied in source selection: the publications focus on rural populations, their relationship with conventional medicine is not documented, and they originate from Central-Eastern Europe. The gathered data were systematically analysed with respect to the plant parts utilised, the methods of preparation, and the specific ailments or diseases for which the plant was employed. Since the aim of the study was to demonstrate the diversity of common applications of dandelion’s raw materials in therapies, the regional diversity was not included in the discussion. Łuczaj [28] and Kujawska et al. [21] pointed out the problems with the correct nomenclature of plant species in ethnographic research. Kluk [2] wrote that “Peasants commonly use the name ‘mniszek’ (dandelion) for all similar plants that have their own latex “. In the present study, the analysis of plant species in the cited ethnographic sources was conducted to exclude species that were referred to with the same name but classified as separate taxa, for example, *Sonchus* spp. [28]. The manuscript was based on printed sources, but especially in the case of Polish-language sources, only a small number of them have been published online. Therefore, this work was created to make this unique knowledge available to a broader audience of international readers. The medicinal use of dandelion is presented from a historical perspective, dividing it into the following periods: 18th century (no earlier works were found), this period covers the period until the fall (1795) of the Polish–Lithuanian Commonwealth, a state that covered a large part of the studied area; the 19th century (domination of the Russian Empire); the period from the beginning of the 20th century until the end of World War II; the period from World War II to the fall of communism (1989); and the period of democratic rule after 1990. The basis for qualifying a publication for a given time period was the date of publication; however, in the case where the time from which the obtained sources originated was clearly stated, such work was included in this period, e.g., Kujawska et al. (2016) [21], a work based on materials collected by Adam Fischer (d. 1943). Regarding the nomenclature of dandelion’s raw materials and formulations, the term ‘latex’ was used with reference to the ‘juice’ or ‘milk’ mentioned in the analysed literature, leaking from damaged plant tissues. Similarly, the term ‘syrup’ was used as a formulation made from dandelion inflorescences with sugar, sometimes with the addition of water, named ‘syrup’, ‘honey’, or ‘jam’ in the sources of the literature.

In the present study, we also tried to refer to the diseases or their symptoms mentioned in the historical sources by the current medical nomenclature, based on the ‘Ethnological Atlas of the human body and diseases‘ [29] and ’Medical vocabulary of Stafan Falimirz‘ [30]. However, in some cases, especially those related to abdominal organs, it was uncertain or even impossible to link the symptoms mentioned in the former literature (commonly pain and colic) with certain diseases [31,32].

## 3. Results

**Figure 1:**
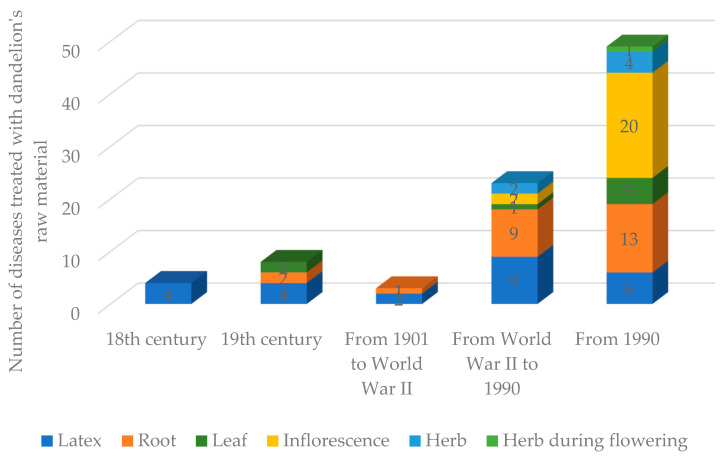
Number of medicinal uses of dandelion’s raw materials in historical periods found in cited references.

Common dandelion is a well-known plant recognised by the local communities of Central-Eastern Europe as a weed but also as an herb. The health-promoting properties of dandelion were known and used in the therapy of many diseases by rural communities since at least the 18th century (Table 1 and Table 2 and Figure 1). The general number of diseases treated with dandelion’s raw material and formulations increased constantly, including several dozen uses at the end of the discussed period (Table 1). Initially, in the 18th century, latex was commonly used, but since the 19th century, information about the medicinal use of leaves or roots had been discussed in the analysed literature. On the other hand, sources published after the Second World War contained information on the use of herbs and inflorescences, and those published after 1990 contained information on the utilization of herbs with inflorescences (Table 2 and Figure 1). Latex and roots were used for the treatment of most of the mentioned diseases in decoction and syrup, using inflorescence as a raw material (Table 2). However, the choice of the treatment procedure should be individual. Special formulations were recommended in the case of only three, two, and even individual diseases (Table 2). The rural population of the investigated regions considered dandelion an effective remedy for internal diseases [33], while populations native to the Balkans used syrup obtained from inflorescences as a panacea [34,35]. During the 18th to 20th centuries, many communities used dandelion for the treatment of dermatological, digestive, respiratory, and urinary diseases [6,14,18,21].

### 3.1. Respiratory System Diseases

The populations native to Central-Eastern Europe exploited dandelion’s herb in the treatment of respiratory diseases, such as asthma, flu, cold, fever, lung diseases, and the infections of the upper respiratory tract, mainly coughing. The syrup, commonly prescribed in these cases, was prepared by sprinkling the collected inflorescences with sugar and, in some cases, boiling it with lemon. The inflorescence syrup was used for the treatment of throat ailments, cough, hoarseness, cold, runny nose, flu, shortness of breath, bronchitis, pneumonia, and even tuberculosis. Sometimes, the syrup was added to tea, for example, to treat flu. The colds were also cured with fresh inflorescence. The infusion of dried inflorescences was used in the case of cough, while the infusion of leaves and roots was used to cure persistent cough. Latex was recommended for dyspnoea, and the wine made of inflorescence for fever. Leaf extract was considered an effective drug against fever, and the root decoction was prescribed for diseases of the upper respiratory tract (Table 3). References from other regions also contained information that dandelion inflorescence syrup was effective in treating the diseases of the respiratory system. In Italy, it was recommended for coughing [73], similarly to Slovenia [74], Serbia [75,76], and Croatia [77]. In Serbia, dandelion inflorescence syrup was also used to treat pulmonary ailments [75] and bronchitis [76]. In Kosovo, an infusion of inflorescences was prescribed to cure respiratory inflammation and the infusion of leaves in the case of lung disorders, such as bronchitis [78,79].

### 3.2. Digestive System Diseases

Dandelion was considered an effective herb for the treatment of internal diseases among rural populations of the analysed area [33]; for example, abdominal pain was alleviated with infusions or tinctures made of its leaves and inflorescences. The data showed that the kind of formulation was of great importance in alleviating the symptoms of digestive system diseases. An infusion of dandelion roots and leaves was used against stomach disorders, but the decoction of these raw materials, as well as the latex and inflorescence syrup, was used against liver ailments. Dandelion root decoction was also a remedy for gall bladder ailments (Table 3). The available data relevant to the use of the herb in the treatment of digestive system diseases collected from various regions broadened the scope of knowledge in this field. For example, to treat stomach pain, the infusion of dandelion inflorescences had been used in Kosovo [78], the herb in the Venetian Diaspora in Eastern Romania [80] and Bolivia [81], and a decoction of the roots in North America [82]. Liver ailments were treated with dandelion’s roots in Himachal Pradesh [83], with a decoction of leaves in the Tuscan Archipelago [84], with an infusion of leaves in Spain [85], and with an infusion of entire plants in Bolivia [81] and Syria [86]. The infusion of whole plants was also used in Bolivia [81] and Syria [86] against biliary ailments.

Dandelion was used in digestive problems, and, for example, the infusion of the roots was drunk in case of poisoning. The infusions of roots, leaves, or fresh leaves were considered a remedy for problems with defecation. The infusion of roots or the wine of inflorescences induced vomiting (Table 3). Dandelion was used as a laxative in Italy [73] and Pakistan [87]. North American Iroquois made a decoction of inflorescences and leaves as a laxative agent, while the infusion of roots was used to induce vomiting [82].

In Central-Eastern Europe, the infusion of the herb was used in the form of baths and compresses against haemorrhoids (Table 2 and Table 3). To alleviate these ailments, dandelion was also used in Italy [88] and in Croatia [89]. Probably due to the signature doctrine attributed to Paracelsus, the determined colour of the dandelion inflorescence was considered to be effective in jaundice treatment; this involved rubbing latex on the face or drinking the infusion of the roots (Table 3). The latter use was also reported in the British Isles [90], Pakistan [91], India, Nepal, and China [92].

### 3.3. Dermatological Diseases

The literature sources from Central-Eastern Europe contain information that dandelion latex or compresses made of inflorescences were used in the treatment of many dermatological diseases, including common warts and boils. Latex was also recommended in the cases of erysipelas and purulent skin infections (Table 2 and Table 3). The healing properties of dandelion reflect its Polish regional names, for example, ‘brodawnik’ or ‘kurzajk’ meaning ‘wart’ [16,17,18]. Similarly, latex was used to remove warts in the British Isles [90,93], Italy [94], and Spain [95]. Moreover, in folk medicine, dandelion was considered to be effective in preventing the formation of dark spots on the skin [75]. In Italy, the decoction of whole plants was recommended for the treatment of skin inflammations [88]. In Himachal Pradesh, the leaf compresses were used as a poultice against swelling and boils [83].

### 3.4. Urinary System Diseases

Dandelion was commonly used in the treatment of urinary system among populations native to Central-Eastern Europe. In the case of kidney disease, the herb or inflorescences were used to make a drink, while the dandelion root decoction was recommended to patients suffering from kidney stones, cystitis, and ascites (general swelling; Lat. hydrops) (Table 3). The diuretic properties of dandelion were recognised in many countries, although different raw materials or formulations were used; for example, the following infusions were reported: leaves in Bolivia [96], whole plants in Syria [86], inflorescences in Kosovo [35], and roots in India [97,98]. In Italy, for this purpose, leaves, inflorescences, or roots were eaten raw or applied as decoction or syrup [73,84,99]. Among the raw materials and formulations recommended for kidney diseases, the following were used: roots in Himachal Pradesh [83] and North America [82], aboveground parts in Bolivia [81], and the infusion of leaves in India [98], Bolivia, and Peru [95].

### 3.5. Pain Treatment

In Central-Eastern Europe, the common dandelion was used to mitigate pain of different origins. Leaf and inflorescence infusions or tinctures were recommended as a remedy for headache, and latex was recommended for tooth or ear pain (Table 3). This application of dandelion herb and formulations was also reported in the literature from the British Isles [90], Balkans [78,100], and North America [82]. In Central-Eastern Europe, fresh inflorescences, as well as tinctures and decoctions of inflorescences and whole plants, were applied topically to relieve pain (Table 3). For this purpose, an infusion of the entire plant was also used in Syria [86] and compresses of roots in Pakistan [87].

Latex from the above-ground parts of dandelion was applied to relieve back pain, while an infusion of roots or inflorescence was prescribed to treat rheumatism (Table 3). Herb was also used for this purpose, as Talko-Hryncewicz [47] described, ‘When a sick person is seated, naked, on a large stone, in the open air in summer, and in a cottage in winter, they lavage his body with a decoction of mullein (*Verbascum* sp.), violets (*Viola* sp.) and dandelion’. Data from the literature also indicated that rheumatism was treated with dandelion herb by people native to the British Isles [90], Syria [86], and North America [82].

### 3.6. Ocular Diseases

The literature related to the folk medicine of Central-Eastern Europe contains contradictory opinions on the effectiveness of dandelion in the treatment of ocular diseases. Some authors indicated that dandelion latex could cause vision loss or night blindness when it enters the eye, whereas others indicated that latex could be used in the treatment of blindness (Table 3). The external application of dandelion latex in cases of eye disease was also mentioned by Kluk in the 18th century [2]. Furthermore, the Iroquois communities of North America Iroquois [82] and those in the British Isles [90] used dandelions’ herb in former ophthalmology.

### 3.7. Cardiovascular System Diseases

There are a few references that common dandelion was an effective remedy in the treatment of cardiovascular disease. Szot-Radziszewska [61] mentioned the use of dried herbs in the case of hypertension, while Kąś [10] provided information on the application of inflorescences to support cardiovascular capacity. In terms of references outside the study region, dandelion was a remedy in the treatment of hypertension in Croatia [89] and in Italy [73], while in Pakistan, the infusion of roots and inflorescences was a remedy applied in cases of heart diseases [82,90,91].

### 3.8. Obstetrics and Gynaecology

A root decoction was commonly recommended for women suffering from abdominal pain (Table 3). Köhler [46] mentioned the use of dandelions to induce a miscarriage. Data from the literature from other regions also provided information on the use of dandelion in relieving menstrual pain [78,82] and in cases of irregular menstruation [101].

### 3.9. Others

The population of Central-Eastern Europe considered the dandelion herb to have beneficial effects on the human body. The herb infusion was drunk to strengthen the body, while the syrup of raw or fermented inflorescence was used to improve the immune system, clean the body, and provide vitamins and minerals (Table 3). Kluk [2] wrote, “This white bitter juice is taken internally in the spring to clean”. The mentioned strengthening and cleansing effect of dandelion did not go unnoticed by other rural communities. For this purpose, leaf salads were eaten in Bolivia [81] and by the Russian community in Germany [102]. In Italy, leaf decoction or the syrup of inflorescences was used [103], while the syrup of inflorescences was used in Slovenia [74]. In Central-Eastern Europe, an infusion of dandelion herb was recommended in cases of diabetes (Table 3), and, moreover, sources from other countries and regions, such as the British Isles [90], Mexico [104], Bosnia and Herzegovina [101], Pakistan [82], and Syria [86], contain similar information. Sõukand et al. [13] reported that in the Liubań region (Belarus), inflorescence syrup as well as raw roots were recommended in cancer cases (Table 3), and similar references originate from Slovenia [74]. The anticancer activity of the dandelion herb has been reported in sources from the British Isles [90] and Mexico [105]. Dandelion was a traditional wound-healing plant not only among the populations of Central-Eastern Europe but also the British Isles [90], Bulgaria, Serbia [106], and India [83,107].

The specificity of Polish folk medicine was the application of dandelion latex in the curation of patients with symptoms of rabies (Table 3). Furthermore, wet leaf dressings were used for herpes zoster and chickenpox (Table 3), but this usage seemed to be limited only to the mentioned regions.

### 3.10. Cosmetics

Common dandelion and its formulations have been widely used as natural cosmetics. The infusion of herbs was used in hair care to prevent hair loss and in skin care for anti-ageing, but in this regard, there is no exact information about the type of raw material or formulation used. Inflorescences and latex were effective in bleaching freckles (Table 3). In Belarus, the decoction of dried dandelion roots was valued as a recreational tea [13]. Information about the use of dandelion root decoction for slimming purposes comes from the Lublin region in Poland (Table 3), while in Syria, the whole plant was used for the same purpose [86].

### 3.11. Veterinary Medicine

The references from Central-Eastern Europe contain selective information on the use of dandelions in veterinary medicine. According to common opinion, dandelion was the favourite food of farm animals, including cows, horses, pigs, rabbits, and ducks (Table 4). It is also known to be used as a purification factor, contributing to wound healing by removing skin-penetrating parasites from livestock. The cattle were fed a decoction of roots as a remedy for flatulence and as a lactogenic. Information on its use as feed for livestock also comes from other regions of the world [83,91,108,109]. In addition, a figurative role was referred to dandelion inflorescences in folk culture. As an example, during Pentecost Sunday, cows were decorated with dandelion’s wreaths to ensure breeding success [110].

## 4. Conclusions

Due to its broad distribution, the common dandelion was an easily available herbal material in Central-Eastern Europe. The information collected indicated that this plant was widely used in folk medicine, and since 1990, the number of dandelion-cured diseases has systematically increased, covering dozens of ailments. Dandelion’s raw materials and formulations were used to treat respiratory, digestive, urinary, cardiovascular system, dermatological, and ocular diseases; they were also used in pain treatment and in obstetrics and gynaecological disorders. Furthermore, dandelion was included among herbs with a beneficial effect on the human body as an immunostimulant and strengthening agent, as well as a cleansing agent. Furthermore, the present study showed a hitherto unknown possibility of using dandelion leaf compresses in the treatment of herpes zoster, chickenpox, and rabies, as these applications appeared to be limited only to the investigated regions. In traditional folk medicine, special attention was paid not only to the raw materials used, including roots, leaves, inflorescences, herbs, or latex, but also to the formulations applied internally, such as infusions, decoctions, syrups, and wine, or externally, such as compresses. This work also points to the individual approach in the utilisation of dandelion raw materials because the selection and preparation methods were specific to disease entities. In the period analysed, there are no reports on the use of the whole plant in folk medicine, while contemporary pharmacognosy indicates the possibility of using a root infusion or decoction of roots together with the herb [114]. The sources gave an ambiguous answer on the healing effect of a dandelion on eyesight because it was found that after latex use, people regained their eyesight, but putting this juice into the eye may even cause its loss. Therefore, phytochemical analysis and determination of the pharmacological effects of dandelion latex can be a very interesting prospect for further phytochemical studies. Furthermore, based on the analysis of the data from the literature, it can be assumed that the common dandelion was recognised as a very valuable feed for animals, suggesting the possibility of using it as a feed supplement in livestock farming.

The data collected reflect the state-of-knowledge of the communities of Central-Eastern Europe concerning the dandelion’s healing properties during the investigated period. Scientific publications published in recent years confirmed the healing properties of common dandelion, indicating the legitimacy of research in this area. Furthermore, the experience of rural communities may indicate the directions for further phytochemical and pharmacological research.

## Figures and Tables

**Table 1:** Medicinal use of dandelion in historical periods.

Period	Medicinal Use
18th century	Body-cleansing agent [2], in ocular disease treatment [2], against wounds and ulcers [2].
19th century	Analgesic agent for earaches [36], toothaches [37], and during tooth eruption in children [38]. Antiswelling agent (*hydrops*) [39]. Vomiting [40] and laxative agent [41]. Treatment of ague (*febris flava*) [42], jaundice [41], vision loss [43], blindness [37], shortness of breath [36], and rheumatism [41], against warts [44,45] and rabies [37]. Aborifacient agent [46].
From 1901 to World War II	Antiswelling [21] and vomiting agent [47]. In cold [21], jaundice [21], and liver disease treatment [21,48]. In treatment of vision loss [21] and night blindness (*nyktalopia*) [15]. Against warts [15].
From World War II to 1989	Analgesic agent for earaches [36,49], toothaches [49], stomachaches [6,50], and abdominal pain in women [50]. Treatment of stomach [50], liver [6,51], biliary disorders [6], and haemorrhoids [51]. Antiswelling [49], constipation [50], and vomiting agent [49]. In erysipelas [49], jaundice [6,52], and vision loss treatment [6,49]. In bladder inflammation [50]. In diseases of the upper respiratory tract [6], including persistent cough treatment [50,51]. Against skin problems [6], including warts [6,17,49,52] and freckles [6,49]. Against rabies [49,52].
After 1990	Analgesic agent [13] in earache [53], toothache [54], stomachache [13], and back pain [55]. Against upper respiratory tract diseases [56], including asthma [54], pulmonary asthma [57], bronchitis [13], rhinitis [58], sore throat [13], hoarseness [10,59], shortness of breath [9,10], pneumonia [60], and tuberculosis [13]. Antitussive [8,10,12,56,59,61,62,63], body cleansing [10], immunostimulant [10,13,61], and recreational agent [13], source of vitamins [13]. Against skin problems [53,54], including wounds [64], mycoses [65], black spots [66], freckles [55,66,67], warts [53,55,58], eczema [65], hair loss [55], to refresh facial skin [18]. Against digestive diseases, including liver diseases [8,33,55,61,68], lung diseases [56], biliary ailments [68], and jaundice [69] and as laxative [68]. Vomiting agent [10,18]. To improve appetite and digestion [68], supporting slimming [12], against poisoning [70]. In cancer treatment [13]. Antipyretic in cold [8,10,13,61] and flu treatment [10]. In diabetes treatment [55]. In vision loss treatment [18,54,61]. Antiswelling [71] and diuretic agent [68,72]. Against kidney stones [13], hypertension [61], herpes zoster [64], rabies [54], and rheumatism [65].

**Table 2:** The use of dandelion in folk medicine of Central-Eastern Europe.

Part of Plant	Raw Material/Preparation	Medicinal Use
Herb	Dried herb, infusion	Against hypertension [15] and as immunostimulant [61]
	Extract	Against bladder inflammation [50]
	Infusion	In liver diseases [51,55], kidney diseases [55], haemorrhoids [51], diabetes [55], suspending hair loss [55]
	Tincture	Analgesic [13]
Inflorescence		In stomach [66] and heart diseases [61]; cosmetic to treat black spots [66] and freckles [49,55,67]
	Decoction	In kidney diseases [13], as an analgesic [13]
	Dried	Antitussive [56]
	Wine	Antipyretic [10], immunostimulant [13], causing vomiting [10]
	Fresh	Antipyretic [13], analgesic [13]
	Infusion	Antitussive [51], in stomach ache [50], rheumatism [65]
	Syrup	Antitussive [8,10,12,59,61,62,63], antipyretic [8,13,61], in hoarseness [10,59], shortness of breath [9,10], rhinitis [58], liver diseases [61], sore throat [13], bronchitis [13], tuberculosis [13], pneumonia [60], cancer [13], as immunostimulant [10], as a source of vitamins [13],
	Tincture	In stomachache [13], analgesic [13], skin treatment after cupping [33]
	Wraps	Against warts [53]
Herb during flowering	Crushed herb	In skin diseases [53]
Leaf	Compress	Against herpes zoster/chickenpox [64]
	Decoction	In stomach diseases [46], hair care [13]
	Fresh	Laxative [41], wound treatment [64]
	Infusion	Against constipation [50], persistent cough [50], and stomach ache [50]
	Tincture	In stomach ache [13], ague (Lat. *febris flava*) [42]
Latex		Analgesic in back pain [55], earache [36,49,53], toothache [37,49]; body cleansing [2]; in eye diseases [2], including night-blindness (Lat. *nyktalopia*) [15] and vision loss [6,18,49]; in jaundice [21,41,69]; in liver diseases [8], in rabies [49,52], in erysipelas (Lat. *erysipelas*) [49], in shortness of breath [36], skin problems [6]: wounds [2], mycoses [65], freckles [6,49,55], and warts [6,15,17,44,45,49,52,55,58]
Root		In biliary ailments [68], mild laxative action [68], increasing appetite and improving digestion [68], as diuretic [68], in liver diseases [68]
	Coffee substitute	In kidney stones [13], as recreational tea [13]
	Decoction	In biliary ailments [6], as diuretic (edema) [72], generalised swelling (*hydrops*) [21,39,49,71], in jaundice [6], in liver disease [6], abdominal pain in women [50], slimming [12], in stomach ache [6], in stomach diseases [49], in upper respiratory tract diseases [6], vomiting [49]
	Extract	Inflammation of the bladder [50]
	Fresh	Cancer [13]
	Grated root	Eczema [65]
	Infusion	Constipation [50], poisonings [70], rheumatism [65]
	Scraped and boiled in milk	Vomiting [18,40]

**Table 3:** The use of dandelion in folk medicine.

Symptoms/Disease Entity	Part of Plant *	Raw Material/Preparation	References
**Respiratory system diseases**			
Lung diseases			[56]
Diseases of the upper respiratory system	R	Decoction	[6]
			[56]
Cough	I	Syrup	[8,10,59,61,62,63]
		Infusion	[51]
		Dried	[56]
		Syrup	[12]
Persistent cough	L	Infusion	[50]
	R	Infusion	[50]
Sore throat	I	Syrup	[13]
Hoarseness	I	Syrup	[10,59]
For breathing problems	I	Syrup	[9,10]
	Lx		[36]
Pulmonary asthma			[57]
Cold	I	Syrup	[8,13,61]
		Fresh	[13]
		Drink	[21]
Flu	I	Tea with honey and dandelion	[10]
Fever	I	Wine	[10]
Ague (łac. *Febris flava*)	L	Macerate	[42]
Rhinitis	I	Syrup	[58]
Bronchitis	I	Syrup	[13]
Pneumonia	I	Syrup	[60]
Asthma			[54]
Tuberculosis	I		[13]
**Digestive system diseases**			
Liver disease	R	Decoction	[6]
			[68]
	H	Infusion	[51,55]
	Lx		[8]
	I	Syrup	[61]
			[21,33,48]
Stomach disease	R	Decoction	[49]
	L	Decoction	[46]
	I		[66]
			[54]
Stomach ache	L	Infusion	[50]
		Tincture	[13]
	R	Decoction	[6]
	I	Tincture	[13]
	I	Infusion	[50]
Biliary ailments	R		[68]
		Decoction	[6]
Vomiting	R	Decoction	[49]
	R	Scraped and boiled in milk	[18,40]
	I	Wine	[10]
		Cooked	[47]
Constipation	R	Infusion	[50]
	L	Infusion	[50]
Mild laxative	R		[68]
Laxative	L	Fresh	[41]
Poisoning	R	Infusion	[70]
Haemorrhoids	H	Infusion, baths, compresses	[51]
Jaundice	R	Decoction	[6]
	Lx		[20,41]
	S	Rubbing the face	[52,69]
Increasing appetite and improving digestion	R		[68]
**Dermatological diseases**			
Skin diseases	HF	Crushed	[53]
	Lx	Squeezed out of the plant stem	[6]
Warts	Lx	Fresh	[6,15,17,44,45,49,52]
			[54]
	Lx		[15,55]
	Lx from leaves	Lubrication	[58]
	I	Wraps	[53]
Eczema	R	Mixture of grated root and honey	[65]
Mycoses	Lx		[65]
Erysipelas	Lx		[6]
**Urinary system diseases**			
Kidney diseases	H	Infusion	[55]
	I	Decoction	[13]
Kidney stones	R	Coffee substitute	[13]
Diuretic	R		[68]
Inflammation of the bladder	H	Extract	[50]
	R	Extract	[50]
Diuretic (edema)	R	Decoction	[72]
Generalised swelling (łac. *hydrops*)	R	Decoction	[21,39,48,71]
**Pain**			
Toothache	Lx		[37,48]
			[54]
Earache	Lx		[36,49,53]
Ear diseases			[54]
Pain	I	Fresh, topical application	[13]
		Decoction	[13]
		Tincture, topical application	[13]
	H	Tincture, topical application	[13]
Back pain	Lx		[55]
Rheumatism	R	Infusion	[65]
	I	Infusion	[65]
		Decoctions	[47]
Skin after cupping	I	Tincture	[33]
**Ocular diseases**			
Eye diseases			[54]
Blindness	Lx		[6,18,49]
			[37,61]
Loss of vision		Seizure causes blindness	[21,43]
Night-blindness (*łac. nyktalopia*)	Lx	Seizure causes blindness	[15]
**Cardiovascular system diseases**			
Hypertension	H	Infusion of dried herb	[61]
Heart diseases	I		[10]
**Obstetrics and gynaecology**			
For lower abdominal pain in women	R	Decoction	[50]
Abortifacient agent			[46]
**Other**			
Diabetes	H	Infusion	[55]
Cancer	I	Syrup	[13]
	R	Fresh, eaten	[13]
Herpes zoster/Chickenpox	L	Compresses	[64]
Rabies	Lx		[6,52]
			[37,54]
Wounds	L	Compresses	[64]
Immunostimulant	I	Fermented with sugar	[13]
		Syrup	[10]
	H	Infusion of dried herb	[61]
Body cleansing			[10]
	Lx		[2]
Vitamins	I	Syrup	[13]
Recreational tea	R	Dried coffee substitute	[13]
**Cosmetics**			
Suspending hair loss	H	Infusion; washing	[55]
Hair care	L	Decoction	[13]
Refreshing the facial skin			[18]
Slimming	R	Decoction	[12]
Easier tooth-eruption in children		Chewing	[38]
Freckles	I		[48]
			[46,67]
	Lx		[6,49,55]
Cosmetics to treat black spots	I		[66]

* H—Herb; HF—Herb during flowering; I—Inflorescence; L—Leaf; Lx—Latex; R—Root.

**Table 4:** The use of dandelions’ raw materials in folk veterinary medicine.

Animal	Disease	Part of Plant *	Manipulation and Way of Use	References
	Removing worms from wounds			[111]
Cows	Stomach bloating	R	Decoction	[6]
	Stimulation of milk production	H	Food	[6,10,43,55]
	Good for cows	I	Fresh	[13]
				[7]
Horses	Good for horses	I	Fresh	[13]
Pigs	Fodder	H	Fresh	[13]
		I	Fresh	[13]
		L	Fresh	[13]
				[112]
Goose	Fodder	H		[6]
Ducks	Fodder			[113]
Rabbits	Fodder			[113]
Home animals	Fodder	L	Fresh	[13]

* H—Herb; I—Inflorescence; L—Leaf; R—Root.

## Data Availability

Data sharing is not applicable as the data are secondary data drawn from already published literature. Sources referred to that are not accessible online are available on request from the authors.
